# Is cumulative frequency of mitochondrial DNA variants a biomarker for colorectal tumor progression?

**DOI:** 10.1186/1476-4598-3-30

**Published:** 2004-10-13

**Authors:** Felix O Aikhionbare, Masood Khan, Delicia Carey, Joel Okoli, Rodney Go

**Affiliations:** 1 Department of Medicine, Morehouse School of Medicine, Atlanta, GA 30310, USA; 2 School of Public Health, University of Alabama, Birmingham, AL 35294, USA; 3 Comprehensive Cancer Center, University of Alabama, Birmingham AL 35294, USA; 4 Department of Surgery, Morehouse School of Medicine, Atlanta GA 30310, USA

## Abstract

To examine the relationship between mitochondrial DNA (mtDNA) alterations and colorectal tumorigenesis, we used high-resolution restriction endonucleases and sequencing to assess the mitochondrial genome from three histologic subtypes of colorectal adenomas (tubular = 8; tubulovillous = 9; and villous = 8), colorectal cancer (CRC) tissues = 27, and their matched surrounding normal tissue (MSNT) = 52. The mitochondrial genomes were amplified using 9 pairs of overlapping primers and systematically analyzed by means of high-resolution analysis. DNA fragments showing a shift in banding patterns between the three adenomas, CRC, in comparison to the MSNT were sequenced to identify the mtDNA alterations. A total of thirty-eight germ-line mtDNA variants were observed in this study. Twenty-two of the thirty-eight were identified as mutations and 59% (13 of 22) were silent mutations and one was a 1-bp insertion. Sixteen of thirty-eight were distinct SNPs in flanking regions of the restriction sites and, 6 of the 16 (37%) SNPs were not previously reported. Most of these mutations/SNPs were homoplasmic and distributed in various regions of mitochondrial genes including the 16S and 12S rRNA. Based on our results, mtDNA germline variants increased in prevalence with adenoma CRC progression. To the best of our knowledge, this is the first report to show an increased prevalence of mitochondrial gene variants in CRC tumorigenesis.

## Findings

Colorectal polyps are a frequent occurrence in the general population and adenomatous changes in these polyps are associated with the overwhelming majority of CRC. These adenomas are the precursor lesions of colon cancer [1]. Currently, clinical management of individuals with colorectal polyps is guided by the histology of the lesion [2]. However, the accuracy of an appropriate staging of colorectal polyps progression to cancer continues to confound clinical pathologists as well as surgeons. Alternative strategies, which augment pathological findings such as the identification of molecular markers that are associated with the colorectal tumor progression, may prove to be a useful as a prognostic tool and a preoperative stage-specific evaluation. Recently, there have been reports of the potential use of mtDNA mutations as a biomarker in the solid tumor of other cancers [3-6] due its repaired less efficiently compared with that of nuclear DNA. Also, Mitochondria have been implicated in cancer given their role in apoptosis [7,8] and its vulnerability to mutation due to the close proximity to a major source of reactive oxygen species (ROS). Nevertheless, definitive mtDNA mutations associated with the progression of tumors such as CRC have yet to be established. Given the connection between mitochondria, ROS, and neoplasia, mtDNA from CRC adenoma, cancer tissues, and pathologically determined matched surrounding non-cancerous tissue were screened for variants, which may be used as biomarkers for colorectal cancer progression. We speculated that there is an association between one or more mtDNA mutations in the adenomatous polyps and CRC progression and the cumulative frequencies of such mtDNA mutations may eventually be demonstrated to be an important marker in adenoma colorectal progression.

This study was approved by the Institutional Review Board of Morehouse School of Medicine and the Research Oversite Committee of the Grady Memorial Hospital, Atlanta, Georgia and the University of Alabama at Birmingham School of Medicine, Birmingham, Alabama. Primary fresh frozen and paraffin embedded tissues from 3 histologic subtypes of adenomatous polyps (8 tubular; 9 tubulovillous; and 8 villous), 27 CRC tissues, and histologically MSNT, n = 52 were obtained from the Tissue Procurement Network at the University of Alabama at Birmingham and the Department of Surgery of Morehouse School of Medicine/Grady Hospital. Adenomas were defined by histologic type, degree of dysplasia, and presence of infiltrating adenocarcinoma in adenoma and classified as tubular, tubulovillus, and villous. CRC diagnosis was confirmed by histological examinations of biospied specimens for all patients and pathological tumor staging for these was based on American Joint Committee on Cancer [9]. The mean age of the study subjects was 66.3 ± 5.7 years.

Genomic DNA was extracted from both frozen and paraffin embedded remnant tissues using Tri-Reagent (Molecular, Research Center Inc., Cincinnati, Ohio) and Purgene DNA purification kits (Gentra Systems, Minneapolis, Minnesota) according to the manufacture's protocol. The mtDNA from each tissue sample was amplified by PCR using nine overlapping mtDNA primer pairs as previously described [10,11], which resulted in large PCR products that exclude the possibility that nuclear pseudogenes were amplified. Each PCR product was digested with 14 restriction endonucleases (AluI, AvaII, BamHI, Dde I, HaeII, HaeIII, HhaI, HincII, HinfI, HpaI, MspI, MboI, RsaI, and TaqI) and then subjected to direct sequencing of both sense and anti-sense strands with ABI 3100 Genetic Analyzer to determine the exact nature of new length polymorphisms/mutations detected by restriction analysis. Sequences were compared (BLAST) to the human mitochondrial DNA sequence (Genbank Accession #J01415). The MITODAT database was used to identify mitochondrial genome sequence variants.

The analysis of mtDNA samples by restriction digestion and sequencing of the DNA fragments showed non-predicted banding patterns either as homoplasmic single bandshifts or heteroplasmic multiple bands on the gel. The PCR product data point in overlapping regions were counted only once. However overlapping regions were used as internal controls for the identification of the variants. Results of the high-resolution restriction analyses and sequencing of the entire mitochondrial genome yielded 38 sequence variants (including 16 SNPs from the flanking restriction sites) for the precursor adenomatous polyps and cancer tissues. In no instance was a variant detected in the adenomatous tissue found in the histologically matched adjacent surrounding tissue, indicating that these were germ-line origin.

Based upon our assessment of the mitochondrial DNA from non-cancer, precancerous (adenomatous polyps), and colorectal cancer tissues using a high-resolution restriction analysis, we have identified sequence variants. To our knowledge this is the first assessment of the mitochondrial genome using solely primary tissue rather than cell lines. This is important since tissues in culture can undergo clonal evolution which can distort frequency data. Although, the restriction analyses identified a number of band shifts indicating site gains or losses, these data only indicated that the predicted sequence had changed. They did not identify specific base pair changes. We therefore used the original primers and a series of nested primers to sequence the band shifts in order to identify these specific changes. A total of thirty-eight germ-line mtDNA variants were observed and all mutations/SNPs were considered as germ-line origin since the variants found in the colorectal adenoma polyps or cancerous tissues were also found in the MSNT tissues. Twenty-two of the thirty-eight were identified as mutations and 59% (13 of 22) were mostly silent mutations of T-to-C. or a G-to-A transition, which is consistent with the mutagenic spectra of oxidative, damage [12,13]. Among them, C3316T/A (ND1; Met-to Met/Ile), G2758A (16SrRNA), T2352C (16SrRNA), A4769G (ND2; Met-to-Met), A3759G (ND1;CUN), G5178T (ND2; CUN-to-Met) G7028A (COI; Ala-to-Ala) T7055C/G (COI; Gly-to-Gly) C7498T (S(UCN)), G6260A (COI; Glu-to-Gly), G8251A (COII; Gly-to-Gly) T8784C (ATPase 6; Gly-to-Gly) A8618G (ATPase6; Ile-to-Thr), G9055A (ATPase 6, Ala-to-Thr), A8860G (ATPase 6 Thr-to-Ala), T11641C (ND4; Met-to-Met), A10398G (ND3; Thr-to-Ala), C10400T (ND3; Thr-to-Ala), C12633A(ND5;S(UCN)), C16390T(D-loop), T16519C(tRNA^Val^). However, one was a 9-bp Ins5892C in a non-coding region between MTTY and MTCOI. Some of these mutations are located in ATP synthase genes that are involved in mtDNA genome maintenance and integrity in yeast [14]. Seventy-five percent (39 of 52) of a G8860A (Thr-to-Ala) mutation in ATPase 6 gene was detected among CRC adenomas and cancer tissues, compared to 14% (7 of 52) in MSNT tissues. This mutational spectrum in ATPase gene could lead to a less efficient mtDNA replication and abnormalities as previously suggested by Maximo et al. [15]. Sixteen of thirty-eight were distinct SNPs in flanking regions of the restriction sites and 10 of the 16 (63%) have been reported, and 6 were not recorded in the MITODAT database [#J01415]. The identified SNPs are 3107delG (ND1; Frameshift), T2914G (16SrRNA), A2706G (16SrRNA), A2768G (16SrRNA), T2885C (16SrRNA), C7521T (tRNA^Asp^), 7335insC (COI; Frameshift), G7256A (COI; Asn-to-Asn), T7146A (COI; Thr-to-S(UCN)), G8206A (COI; Met-to-Met), A16325G (D-loop), T16309G (D-loop), G16294A (D-loop), G16266A (D-loop), G16233A (D-loop), 8269-9bp del (noncoding). Worth mentioning is also the high frequency of 16S rRNA gene variants (> 65%) in the mtDNA among CRC tissue versus the < 25% in the precancerous tissues. Mutations in the 16S rRNA gene were commonly found in different cancers, except those of the thyroid [16].

**Figure 1:**
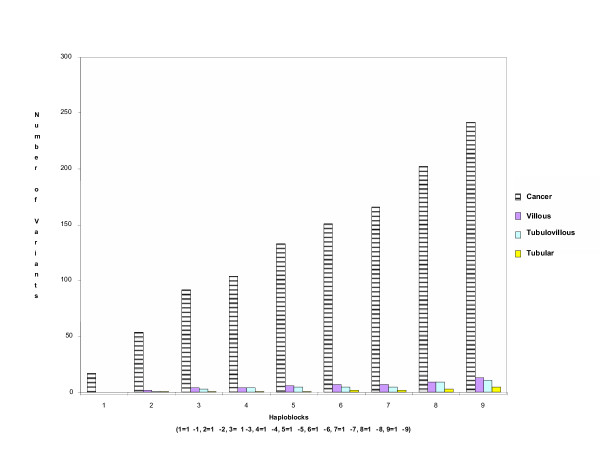
Frequencies of mitochondrial genome variants based on high-restriction analysis in all of the colorectal tumors (tubular, tubulovillous, villous), cancer tissues in each of the 9 primer sets. Numbers on the abscissa represent PCR products obtained using overlapping primers sets 1–9, termed haploblocks (Rank methods; for all comparsion).

We then sought to determine whether or not any of mtDNA variants observed in flanking regions of these restriction site variants may prove to be informative biomarkers. Although, a total of 38 of the mtDNA variants were found, none of the variants appeared to be a marker for a particular adenoma CRC tumor tissue type. Nevertheless, cumulative frequencies of mtDNA variants in the different tissue types resulted in a high prevalence of mtDNA sequence variants in CRC tissue and the trend was for the number of variants to be lowest in the precancerous (Fig. 1), suggesting that this may be a useful approach to distinguishing the progressive stages of CRC adenomas as previously observed in tumour progression in the thyroid [17]. Our sample of CRC adenomas is relatively small and these data must not be overinterpreted at this point. The data however are suggestive and the cumulative frequency approach is currently being followed-up. It is possible that the high frequency of variants in mtDNA in CRC cancer tissues may result from the high rate of mtDNA replication. Furthermore, most of the mtDNA mutations in this study could be a result of mtDNA aggression affected by reactive oxygen species [18-21] and could occur via a slipped replication mechanism [22].

These data should be interpreted cautiously as they are based on small number of sample size. Similar studies have looked at precancerous tissue types [20], however they have not assessed cumulative frequencies of mutations in these same tissues as we have done. Our study is also different in that we did not use cell lines for this work. Moreover, consistent with our findings were reports of cumulative mitochondrial DNA damage in the aging process as well as in cancer [23]. A similar mechanism may be involved in colorectal cancer progression, since age is a risk factor for CRC.

## Abbreviations

MtDNA, mitochondrial DNA; ATP, adenosine triphosphate; CRC, colorectal cancer; MSNT, matched surrounding Normal tissue; SNPs single nucleotide polymorphisms; PCR polymerase chain reaction.

## Authors' contributions

FOA performed few assays while MK performed almost all assays. DC provided the statistical support. JO provided some tissue samples and suggestions for finalization of the manuscript. RG provided some technical supervision to MK and suggestions for finalization of the manuscript. All authors read and approved the manuscript.
